# 5-Lipoxygenase Inhibition Protects Retinal Pigment Epithelium from Sodium Iodate-Induced Ferroptosis and Prevents Retinal Degeneration

**DOI:** 10.1155/2022/1792894

**Published:** 2022-02-23

**Authors:** Jong-Jer Lee, Guo-Ping Chang-Chien, Sufan Lin, Yu-Ting Hsiao, Mu-Chan Ke, Alexander Chen, Tsu-Kung Lin

**Affiliations:** ^1^Department of Ophthalmology, Kaohsiung Chang Gung Memorial Hospital and Chang Gung University College of Medicine, Kaohsiung 833401, Taiwan; ^2^Center for Mitochondrial Research and Medicine, Kaohsiung Chang Gung Memorial Hospital, Kaohsiung 833401, Taiwan; ^3^Super Micro Mass Research and Technology Center, Cheng Shiu University, Kaohsiung 833301, Taiwan; ^4^Center for Environmental Toxin and Emerging-Contaminant Research, Cheng Shiu University, Kaohsiung 833301, Taiwan; ^5^Institute of Environmental Toxin and Emerging-Contaminant, Cheng Shiu University, Kaohsiung 833301, Taiwan; ^6^Department of Neurology, Kaohsiung Chang Gung Memorial Hospital and Chang Gung University College of Medicine, Kaohsiung 833401, Taiwan

## Abstract

Excessive reactive oxygen species (ROS) contribute to damage of retinal cells and the development of retinal diseases including age-related macular degeneration (AMD). ROS result in increased metabolites of lipoxygenases (LOXs), which react with ROS to induce lipid peroxidation and may lead to ferroptosis. In this study, the effect of 5-LOX inhibition on alleviating ROS-induced cell death was evaluated using sodium iodate (NaIO_3_) in the retinal pigment epithelium (RPE) cell line ARPE-19 and a mouse model investigating oxidative stress in AMD. We demonstrated that NaIO_3_ induced cell death in the RPE cells through mechanisms including ferroptosis. Inhibition of 5-LOX with specific inhibitor, Zileuton, or siRNA knockdown of *ALXO5* mitigated NaIO_3_-induced lipid peroxidation, mitochondrial damage, DNA impairment, and cell death in ARPE-19 cells. Additionally, in the mouse model, pretreatment with Zileuton reduced the NaIO_3_-induced lipid peroxidation of RPE cells, cell death in the photoreceptor layer of the retina, inflammatory responses, and degeneration of both the neuroretina and RPE monolayer cells. Our results suggest that 5-LOX plays a crucial role in ROS-induced cell death in the RPE and that regulating 5-LOX activity could be a useful approach to control ROS and ferroptosis-induced damage, which promote degeneration in retinal diseases.

## 1. Introduction

Oxidative stress has been implicated in the pathogenesis of various retinal diseases. Excessive production of reactive oxygen species (ROS) may lead to impairment of retinal cells including retinal pigment epithelium (RPE) cells and vascular endothelial cells. It may also promote development and acceleration of retinal diseases, such as age-related macular degeneration (AMD), diabetic retinopathy (DR), glaucoma, retinal vascular occlusion, and inherited retinal diseases [1].

Various signaling pathways have been proposed to clarify the mechanisms of ROS-induced retinal degeneration. Glutamate is an important neurotransmitter in the retina. However, excessive glutamate triggers calcium overload and subsequently increases mitochondrial ROS, which may prompt retinal ganglion cell death via a mitochondrial-targeting mechanism [2–4]. Treatments that alleviate ROS, such as nuclear factor (erythroid-derived 2)-like 2 gene therapy and spermidine, an endogenous ROS scavenger, successfully protect retinal cells from cell death in experimental models [5–7]. Mechanisms of cell death, including apoptosis, necroptosis, and recently, ferroptosis, have been reported in the research of retinal diseases [8–12]. Iron overload in the neuroretina and RPE may be a contributing factor in the progression of AMD or other diseases involving intraocular hemorrhage [13–15]. Ferroptosis is one of the nonapoptotic forms of programmed cell death, which is characterized by the development of iron-dependent lipid peroxidation [16]. It is regulated by the enzyme, glutathione peroxidase 4 (GPx4), with glutathione (GSH) synthesized from amino acids [17]. The membrane cystine transporter system xc- and GPx4 protect cells from ferroptosis [18]. Toxic substances may develop when superoxide interacts with membrane lipids and produce lipid hydroperoxides through Fenton reaction in the presence of excessive iron or deficiencies in antioxidant defense responses [19].

Both enzymatic and nonenzymatic metabolic pathways, such as cystine deprivation (CD), mevalonate, lipid metabolism, GPx4, iron metabolism, and ferritinophagy pathways, have been reported to be involved in the regulation of ferroptosis [20]. Lipoxygenases (LOXs), a group of enzymes that metabolize arachidonic acid as well as polyunsaturated fatty acids (PUFA), may contribute to lipid peroxidation and induce ferroptosis [21, 22]. Inhibitors of LOX inhibitors, such as 5-LOX inhibitors Zileuton and BWB70C, 12/15-LOX inhibitor baicalin or baicalein, and pan-LOX inhibitor nordihydroguaiaretic (NDGA), have been reported to clear free radicals and therefore decrease the levels of lipid and protein oxidation [23]. Recombinant pigment epithelium-derived factor receptor (PEDF-R) proteins inhibit 5-LOX and protect RPE from ROS-induced cell death *in vitro* [24]. In ARPE-19 cells, docosahexaenoic acid (DHA) suppressed the H_2_O_2_-induced transcriptional upregulation of 5-LOX and proinflammatory hydroxyeicosatetraenoic acids (HETEs) following omega-6 PUFA oxidation [25]. These results imply the role of 5-LOX in lipid peroxidation in RPE cells. However, to the best of our knowledge, no studies have implicated the direct involvement of 5-LOX in RPE or retinal cell death under excessive oxidative stress in *in vitro* or *in vivo* models.

The role of LOXs in retinal or RPE diseases is still poorly understood. In this study, we investigated the role of 5-LOX in ROS-damaged RPE and retina with sodium iodate- (NaIO_3_-) induced cell death in RPE. We used ARPE-19 cells as an *in vitro* model, as well as a murine model of NaIO_3_-induced acute retinal damage, which has been widely used for investigating ROS-induced effects in AMD [26, 27]. Both pharmacological and genetic approaches were used to verify the effect of LOX inhibition on protecting the retina or RPE from regulated cell death such as ferroptosis.

## 2. Materials and Methods

### 2.1. Materials and Reagents

Protein Block (ab156024) was purchased from Abcam (Cambridge, UK). ATP Detection Assay Kit-Luminescence (700410), arachidonic acid (90010), BLX-3887 (27391), deferoxamine (DFO, 14595), Erastin (17754), ferrostatin-1 (17729), JC-1 (15003), Mk-886 (10133), ML355 (18537), necrostatin-1 (11658), RSL3 (19288), Zileuton (10006967), and Z-VAD-FMK (14467) were purchased from Cayman (Ann Arbor, MA, USA). A black 96-well glass-bottom plate was purchased from Cellvis (Mountain View, CA, USA). Cell culture plates including 6-, 12-, and 96-well plates were purchased from Corning (Corning, NY, USA). Cell Counting Kit-8 (CCK-8, CK04) and FerroOrange (F374-10) were purchased from Dojindo (Kumamoto, Japan). Scramble control (SR30004) and the siRNA specific for human *ALOX5* (SR319325) were purchased from OriGene (Rockville, MD, USA). Corn oil (sc-214761) was purchased from Santa Cruz (Dallas, TX, USA). NaIO_3_ (S4007) and tert-Butyl hydroperoxide (tBHP, 458139) were purchased from Sigma-Aldrich (St. Louis, MO, USA). Bodipy C11 (D3861), Dulbecco's Modified Eagle's Medium/Nutrient Mixture F-12 (DMEM/F-12, 12400024), fetal bovine serum (FBS, A3160602), glass-bottom 8-well chamber slides (154534PK), Lipofectamine RNAiMAX Transfection Reagent (13778075), MitoSOX (M36008), M-PER™ Mammalian Protein Extraction Reagent (M-PER, 78501), and T-PER™ Tissue Protein Extraction Reagent (T-PER, 78510) were purchased from Thermo Fisher (Waltham, MA, USA). Quick-RNA™ Miniprep Kit was purchased from Zymo (Irvine, CA, USA).

### 2.2. Cell Culture

The RPE cell line ARPE-19 (ATCC, Manassas, VA, USA) was seeded at densities of 1.2 × 10^6^ cells per well in 6-well plates, 6 × 10^5^ cells per well in 12-well plates, or 5 × 10^4^ cells per well in 96-well transparent plates and grown to full confluence. The cells were cultured in DMEM/F-12 medium supplemented with HEPES (Thermo Fisher), 10% FBS, and 100 U/mL of penicillin-streptomycin at 37°C with 5% CO_2_. Cells used in experiments were within five passages after thawing from liquid nitrogen.

### 2.3. ALOX5 Knockdown with siRNA

To knockdown *ALOX5*, ARPE-19 cells were transfected for 72 h in 24-well plates with three unique 27mer siRNA duplexes specific for human *ALOX5* (OriGene, SR319325, 10 nM), or with scrambled siRNA (OriGene SR30004, 10 nM) as negative control, using the Lipofectamine RNAiMAX Transfection Reagent (Thermo Fisher) according to the manufacturer's protocol. In vitro experiments were carried out 5 days after the start of the transfection procedure. Effectiveness of the *ALOX5* knockdown was examined using western blot analysis for 5-LOX protein.

### 2.4. Cell Viability Assay

Cells were seeded in sterile transparent 96-well plates. The culture media was mixed with 10% (*v*/*v*) CCK-8 reagent (Dojindo) and incubated for 1 h at 37°C in an incubator. The absorption was then measured at 450 nm using the Hidex Sense Microplate Reader (Hidex, Turku, Finland). The cell viability was assessed with ARPE-19 seeded in 6 independently culture wells (*n* = 6) for each treatment condition.

### 2.5. Fluorescence Probes for Oxidative Stress and Intracellular Ferrous Ion

ARPE-19 cells seeded in black glass-bottom 96-well plates were stained with BODIPY C1 (Ex/Em: 488/520 and 590 nm, 5 *μ*M 30 min, *n* = 6 per treatment condition), H2DCFDA (Ex/Em: 488/520 nm, 15 *μ*M, 2 h, *n* = 8 per treatment condition), and MitoSOX (Ex/Em: 520/590 nm, 2.5 *μ*M, 15 min, *n* = 12 per treatment condition) to detect lipid peroxidation, early intracellular ROS, and mitochondrial ROS, respectively. Intracellular ferrous iron was stained with FerroOrange (Ex/Em: 535/575 nm, 1 *μ*M, 30 min, *n* = 12 per treatment condition). Fluorescence intensity was detected using the Hidex Sense Microplate Reader (Hidex). The representative live imaging of BODIPY C11 and H2DCFDA staining was obtained using ARPE-19 cells seeded in 8-well chamber slides with the inverted microscope DMI3000 B (Leica, Wetzlar, Germany).

### 2.6. HPLC Detection of Arachidonic Acid Metabolites

The intracellular component of ARPE-19 cells was extracted using subcellular fractionation buffer (HEPES 20 mM, KCl 10 mM, MgCl_2_ 2 mM, EDTA 1 mM, EGTA 2 mM, DTT 1 mM, and proteinase inhibitor). Then, 100 *μ*L of the sample was fortified with isotopically labeled analogues of arachidonic acid and 5-hydroxyeicosatetraenoic acid (5-HETE) that functioned as isotope internal standards. Next, the sample was acidified with 0.5 mL 1% formic acid aqueous solution and mixed with ethyl acetate solution to extract the method analytes, including arachidonic acid, 5-HETE, and their isotope-labeled analogues. The extract was concentrated to dryness with nitrogen and then reconstituted in 50 *μ*L acetonitrile and 50 *μ*L 0.1% formic acid aqueous solution. The analytes were separated on a Poroshell EC-18 column (Agilent Technologies, Santa Clara, CA, USA) with gradient elution using a mobile phase made of ultrapure water and acetonitrile. Detection was carried out using negative electrospray ionization and multiple reaction monitoring (MRM) in liquid chromatography-tandem mass spectrometry. The concentration of arachidonic acid and 5-HETE was calculated using the isotope internal standard. The data was acquired and processed using Agilent MassHunter Workstation Version 10.1 software (Agilent Technologies). Detailed methodology is described in Supplemental Method S1.

### 2.7. Evaluation of Mitochondrial Function and Integrity

Mitochondrial function and integrity of ARPE-19 cells were evaluated using ATP Detection Assay Kit-Luminescence (Cayman) and JC-1 staining (Cayman), respectively. In the ATP detection, the luciferin in assay reagent was converted to oxyluciferin with light emission that was quantitatively proportional to the amount of intracellular ATP (*n* = 6 per treatment condition). JC-1 staining (1 *μ*M 30 min, *n* = 6 per treatment condition) was performed as per the manufacturer's protocol. Both luminescence and fluorescence were detected using the Hidex Sense Microplate Reader (Hidex).

### 2.8. Animal Care for In Vivo Experiments

Animal experiments were conducted in accordance with the Association for Research in Vision and Ophthalmology (ARVO) Statement on the Use of Animals in Ophthalmic and Vision Research. All procedures involving animals were approved by the Institutional Animal Care and Use Committee of Kaohsiung Chang Gung Memorial Hospital (Kaohsiung, Taiwan). C57BL/6JNarl mice (National Laboratory Animal Center, Taipei, Taiwan) were maintained on 12 h light/12 h dark cycles with *ad libitum* food and water.

### 2.9. NaIO_3_-Induced Acute Retinal Degeneration of a Murine Model

Acute retinal degeneration was induced with intraperitoneal injection of NaIO_3_ (40 mg/kg of body weight) in sterile phosphate-buffered saline (PBS) in 6-week-old male C57BL/6JNarl mice based on the previously published protocols [26, 27]. Mice in the control group were injected with equal volume of PBS. Mice were sacrificed with intraperitoneal injection of Zoletil® 50 (zolazepam and tiletamine, 100 mg/kg body weight) (Virbac, Carros, France) and xylazine (100 mg/kg body weight) (Bayer, Leverkusen, Germany) 24 h, 3 days, and 14 days following NaIO_3_ injection depending on the experimental design. For protecting RPE/retina from NaIO_3_-induced damage, 5-LOX inhibitor Zileuton (20 mg/kg, dissolved in 10% DMSO and 90% corn oil), DFO (100 mg/kg in PBS), or Ferrostatin-1 (5 mg/kg, dissolved in 3% DMSO and 97% PBS) was injected intraperitoneally twice, at 24 h and 15 min before NaIO_3_ treatment.

### 2.10. Cryosections of Mouse Retina

After sacrificing the mice, eyeballs were extracted and fixed with 4% paraformaldehyde (PFA) for 3 h at 4°C, followed by serial dehydration in sucrose (concentration from 10 to 30%) at 4°C, and then embedded in Tissue-Tek® O.C.T. Compound (4583, Sakura Finetek, Torrance, CA, USA). Transverse cryosections (20 *μ*m thick) were cut and mounted onto glass slides for drying and were stored in a -80°C freezer before staining.

### 2.11. Detection of mRNA Expression with Real-Time PCR

The total RNA of mouse RPE/choroid was extracted using the Quick-RNA™ Miniprep Kit (Zymo) and then reversely transcripted with the cDNA Reverse Transcription Kit (Thermo Fisher). Quantitative real-time PCR was performed for mouse *ALOX5*, *SLC7A11*, *GPX4*, *CHAC1*, *CISD1*, *HSPB1*, *CD80*, *TNF*, and *ACTB* using specific primers (Table 1) and SYBR Green qPCR assays using Applied Biosystems™ 7500 Fast Real-time PCR System (Thermo Fisher). Fold changes in mRNA expression were analyzed using the comparative cycle threshold method. *ACTB* was used as the housekeeping gene for the analysis of target gene expression.

### 2.12. Western Blot Analysis

ARPE-19 cells (*n* = 3) and mouse RPE/choroid tissue of one eyeball (*n* = 3) were lysed in M-PER and T-PER (both from Thermo Fisher), respectively. All samples were mixed with a protease/phosphatase inhibitor cocktail (Abcam), and protein concentration was measured using Coomassie protein assay. Protein samples (10-15 *μ*g) were prepared with 4x Laemmli Sample Buffer with 1.25% (*v*/*v*) 2-mercaptoethanol and denatured at 90°C for 5 min. Equal amounts of protein were separated on an SDS-PAGE gel and subsequently transferred to PVDF membranes with a wet transfer system (Hoefer, Holliston, MA, USA). The PVDF membrane was then blocked with Protein Block (Abcam). Next, the membranes were incubated with primary antibodies for 5-LOX (ab169755, Abcam), GPx4 (ab125066, Abcam), succinate dehydrogenase complex, subunit A, flavoprotein variant (SDHA, #11998, Cell Signaling, Danvers, MA, USA), human xCT/SLC7A11 (#12691, Cell Signaling), and mouse xCT (NB300-318, Novus, Centennial, CO, USA), diluted in TBST with 5% (*w*/*v*) BSA overnight at 4°C. The membranes were then incubated with HRP-linked secondary antibodies for 1 h at room temperature (RT). The protein bands were visualized with HRP substrate reagent (Thermo Fisher) and detected with an ECL imaging system (Bio-Rad, Hercules, CA, USA). The relative expression of target protein was obtained after normalizing to *β*-actin (ab8227, Abcam) expression.

### 2.13. Immunofluorescence Imaging for In Vitro Studies

For immunofluorescence imaging of intracellular structure, ARPE-19 cells were seeded at a density of 0.8 × 10^5^ cells per well in 8-well chamber slides and fixed with 4% PFA and permeabilized with 0.2% Triton X-100 (in PBS). Following blocking with 5% normal goat serum, the slide was incubated with primary antibody for cytochrome c oxidase subunit 4 (COX-4, ab16056, Abcam), succinate dehydrogenase [ubiquinone] iron-sulfur subunit (SDHB, 10620-1-AP, Proteintech, Rosemont, IL, USA) or 8-hydroxy-2′-deoxyguanosine (8-OHdG) conjugated with Alexa Fluor® 647 (sc-393871 AF647, Santa Cruz) at 4°C overnight. These slides were then incubated with Alexa-conjugated secondary antibody for 60 min at RT for unconjugated primary antibodies. Nuclei were counterstained with DAPI. Images were captured using an immunofluorescence microscope Axio Imager M2 (Carl Zeiss, Oberkochen, Germany). 8-OHdG-stained cells were counted in three 400x power fields (more than 50 cells in each field) per slide. The mean percentages of 8-OHdG-positive cells in each slide were compared among the groups with four independent experiments.

### 2.14. Immunofluorescence Imaging for In Vivo Studies

The primary antibodies used include anti-Iba1 (019-19741, Fujifilm Wako, Osaka, Japan), anti-4-hydroxynonenal (HNE) (HNEJ-2, JaICA, Shizuoka, Japan), and anti-zonula occludens-1 (ZO-1, 61-7300, Thermo Fisher). For flat mount imaging of the RPE monolayer, the eyeball was cut along the equator. After removal of the anterior segment and neuroretina, the eyecup was fixed with 4% PFA for 3 h, blocked with mouse-on-mouse blocking reagent (ab269452, Abcam) for mouse-derived primary antibodies or 5% normal goat serum for the rabbit-derived primary antibodies with 0.3% Triton X-100 in PBS for 1 h at RT. Next, the eyecup was incubated with primary antibodies 4-HNE (*n* = 4, 24 h after NaIO_3_ injection), Iba1 (*n* = 4, 3 days after NaIO_3_ injection), or ZO-1 (*n* = 12 animals, 14 days after NaIO_3_) overnight at 4°C and then with Alexa flour-secondary antibody for 1 h. The eyecup was flat-mounted on a glass slide after eight radial cuts and prepared in mounting medium for immunofluorescence imaging. Fluorescence intensity of 4-HNE was measured in the total surface area of the RPE monolayer under low-power 50x images. The number of Iba1-positive cells was counted in four independent 100 *μ*m × 100 *μ*m square areas, which were chosen about 400 *μ*m away from the optic disc under 100x images. In each 10000 *μ*m^2^ area, the mean counts of Iba1-positive cells were compared between the four retinas in each treatment condition. The integrity of RPE monolayer, hexagonal structure of intercellular junction between RPE cells, in the mouse retina was evaluated with ZO-1 staining. RPE monolayer without clear and regularly aligned hexagonal ZO-1 staining in more than 25% area of the retina (under 200x magnification) was considered destructed. Cryosections of eyeballs were used for TUNEL staining and retinal thickness assessment. TUNEL staining of mouse retinal cryosections was performed with DAPI counterstained nuclei using the manufacturer's protocol (Roche, Basel, Switzerland). TUNEL-positive cells were counted in the region of the retina 200 to 800 *μ*m away from the optic disc. The mean number of cells in three sections from each eyeball was compared among groups (*n* = 8). Retinal thickness was evaluated with DAPI-stained retinal cryosections obtained after 14 days of treatment with NaIO_3_. The average retinal thickness of the three sections from each eyeball 200, 400, 600, and 800 *μ*m away from the optic disc was compared (*n* = 6 in each group). Both flat-mount and retinal-sectional images were obtained using the immunofluorescence microscope Axio Imager M2 (Carl Zeiss). Fluorescence intensities in the images were analyzed for quantitative comparison between groups with ImageJ.

### 2.15. Statistical Analyses

The differences between the control and treatment groups were compared using Student's *t*-test for two groups and one-way ANOVA with the post hoc Sidak multiple comparison correction test using GraphPad Prism 9.2.0 (GraphPad Software, San Diego, CA, USA). The error bars in the figures represent standard deviation (SD). Significant significance was set at *p* value < 0.05.

## 3. Results

### 3.1. NaIO_3_ Induces Oxidative Stress and Cell Death That Is Mitigated by Treatment with Ferroptosis Inhibitors in ARPE-19 Cells

**Figure 1:**
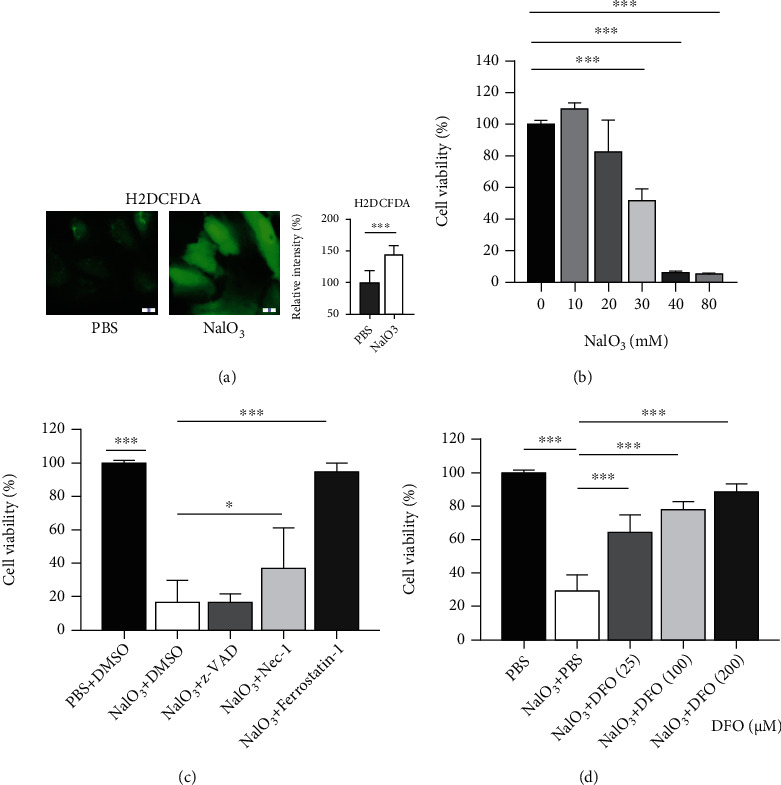
Sodium iodate (NaIO_3_)-induced oxidative stress and cell death, which was mitigated by treatment with ferroptosis inhibitors in ARPE-19 cells. (a) Representative images of cells treated with PBS and NaIO_3_ (35 mM, 2 h) and stained with H2DCFDA (left) and measured using a microplate reader (right, *n* = 8). Scale bar = 10 *μ*m. (b) Ferroptosis induced by NaIO_3_ under different concentrations (*n* = 6 per concentration, 20 h), analyzed by Cell the Counting Kit (CCK-8). (c) Effect of different inhibitors on NaIO_3_-induced cytotoxicity: 33 *μ*M z-VAD (*n* = 6), 40 *μ*M Nec-1 (*n* = 6), and 2 *μ*M ferrostatin-1 (*n* = 6). (d) Effect of different concentrations of iron-chelator deferoxamine (DFO) on NaIO_3_ (35 mM, 20 h)-induced cell death (*n* = 6 per concentration), analyzed by CCK-8 assay. ^∗^*p* < 0.05, ^∗∗^*p* < 0.01, and ^∗∗∗^*p* < 0.001. Abbreviations: H2DCFDA: 2′,7′-dichlorodihydrofluorescein diacetate; Nec-1: necrostatin-1; PBS: phosphate-buffered saline; z-VAD: z-VAD-FMK.

NaIO_3_ treatment induced cell death with more than 50% decline in ARPE-19 cells at NaIO_3_ concentrations higher than 30 mM (Figure 1(a)). Lethal concentration of NaIO_3_ (35 mM, 20 h) induced rapid elevation of intracellular ROS in ARPE-19 cells, which was detected with H2DCFDA staining 1 h after NaIO_3_ treatment (Figure 1(b)). To identify the underlying mechanism of cell death induced by NaIO_3_, inhibitors for different pathways including apoptosis (z-VAD-FMK, 33 *μ*M, pretreatment 8 h), necroptosis (necrostatin-1, 40 *μ*M, pretreatment 8 h), and ferroptosis (Ferrostatin-1, 2 *μ*M, pretreatment 8 h) were used to protect cells from NaIO_3_-induced loss in cell viability. We found that pretreatment with ferroptosis inhibitor, ferrostatin-1, partially alleviated NaIO_3_-induced cell death (Figure 1(c)). Furthermore, 8 h pretreatment with iron-chelator DFO also prevented NaIO_3_-induced cell death in ARPE-19 cells (Figure 1(d)). These results suggest that ferroptosis may be one of the major mechanisms of cell death induced by NaIO_3_.

### 3.2. Inhibition of 5-LOX Reduces NaIO_3_-Induced Cell Death and ROS Production

**Figure 2:**
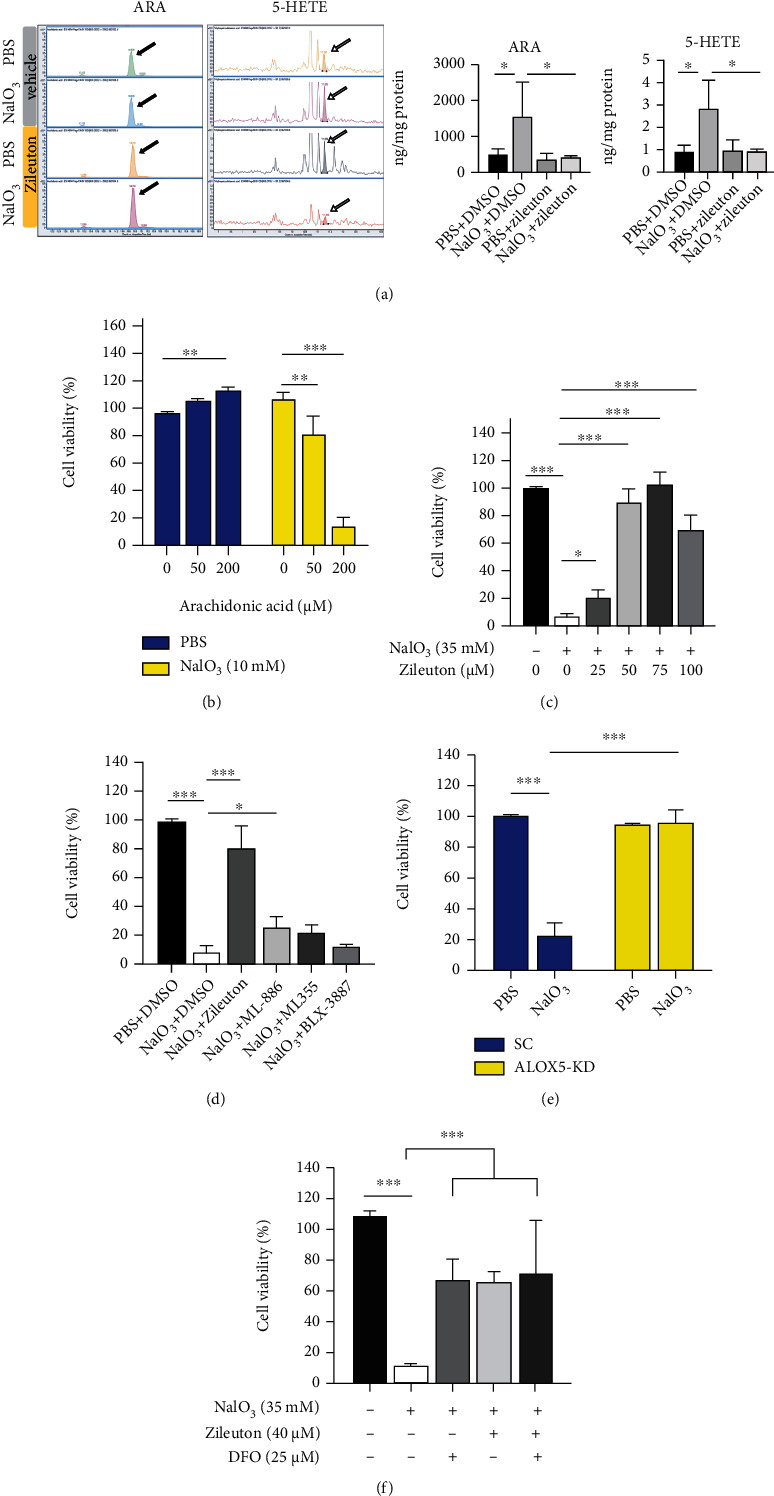
Inhibition of lipoxygenase-5 (5-LOX) protected ARPE-19 cells from NaIO_3_-induced cell death by ferroptosis. (a) Arachidonic acid (ARA, black arrow) and 5-hydroxyeicosatetraenoic acid (5-HETE, white arrow) were detected with HPLC-MS/MS analysis. CCK-8 was used to analyze cell viability following treatments (b–f). Cell viability of ARPE-19 following treatment with (b) nonlethal concentration of NaIO_3_ (10 mM, 20 h) and different concentrations of arachidonic acid, (c) toxic NaIO_3_ (35 mM, 20 h) and different concentrations of 5-LOX inhibitor Zileuton (*n* = 6 per treatment condition), and (d) other types of LOX inhibitors (75 *μ*M Zileuton, 15 *μ*M Mk-886, 3 *μ*M ML355, 500 nM BLX-3887, *n* = 6 per treatment condition). (e) The effect of NaIO_3_ (35 mM, 20 h) with *ALOX5* knockdown-ARPE-19 cell. (f) Effect of combined treatment with suboptimal concentrations of Zileuton (40 *μ*M) and DFO (25 *μ*M) on NaIO_3_ (35 mM, 20 h)-induced ferroptosis. Abbreviations: HPLC-MS/MS: high-performance liquid chromatography with tandem mass spectrometry; PBS: phosphate-buffered saline; sc: scramble; KD: knockdown.

**Figure 3:**
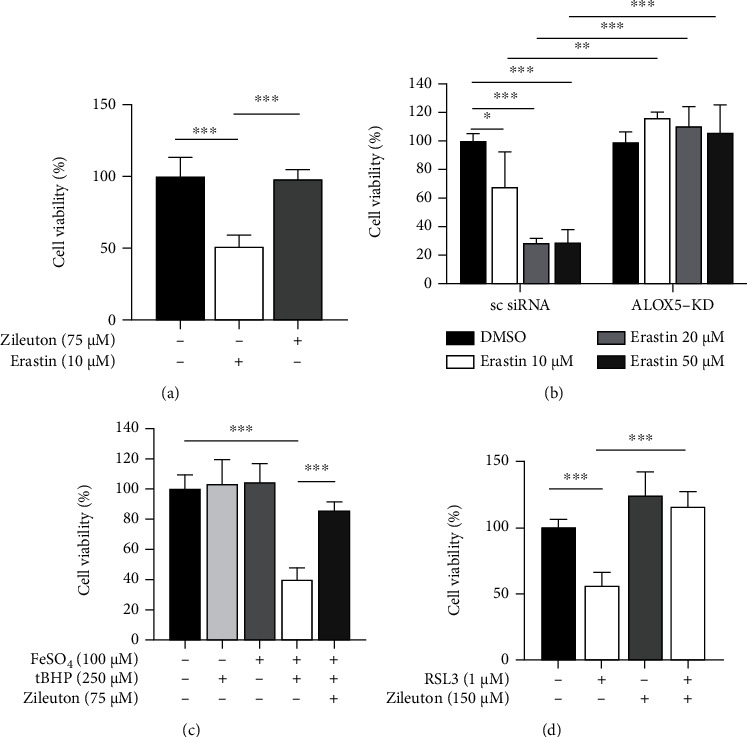
Inhibition of lipoxygenase-5 (5-LOX) protected ARPE-19 cells from cell death induced by conventional ferroptosis inducers. Cell viability of ARPE-19 cells following ferroptosis induction with (a, b) Erastin (24 h, *n* = 6 per treatment condition), (c) combined treatment with ferrous ion (FeSO_4_ 100 *μ*M, 8 h) and tBHP (250 *μ*M, 8 h, *n* = 6 per treatment condition), and (d) RSL3 (1 *μ*M, 20 h). Abbreviations: tBHP: tert-Butyl hydroperoxide.

Oxidative stress can induce the mobilization of arachidonic acid from membranous structures in the cells [28]. Arachidonic acid can be metabolized by lipoxygenases to produce 5-hydroperoxyeicosatetraenoic acid (5-HPETE), which is associated with lipid peroxidation and ferroptosis [29]. HPLC-MS/MS analysis showed that NaIO_3_ (35 mM, 15 h) induced upregulation of arachidonic acid and its 5-LOX-dependent stable metabolite 5-HETE in cell lysates of ARPE-19 cells (Figure 2(a)). Other arachidonic acid-associated metabolites, such as 8-iso prostaglandin F2*α* and prostaglandin F2*α*, were not detected. Externally derived arachidonic acid increased the susceptibility to nonlethal NaIO_3_ (10 mM, 20 h)-induced cell death when compared with cells cultured in serum-free medium (Figure 2(b)). Pretreatment with the 5-LOX inhibitor Zileuton for 8 h protected ARPE-19 cells from NaIO_3_-induced cell death and maintained cell viability (Figure 2(c)). In contrast, 5-LOX activating protein inhibitor Mk-886 (15 *μ*M), 12-LOX inhibitor ML355 (3 *μ*M), and 15-LOX inhibitor BLX-3887 (500 nM) had limited or no effect on NaIO_3_-induced cell death (Figure 2(d)). We further verified the role of 5-LOX in ARPE-19 cells lacking 5-LOX due to knockdown of *ALOX5* with siRNA (*ALOX5*-KD). *ALOX5*-KD cells were resistant to NaIO_3_-induced cell death when compared with cells treated with scrambled siRNA (Figure 2(e)). Additionally, the combination of Zileuton (40 *μ*M) and DFO (25 *μ*M) at suboptimal concentrations did not exert an additive effect in protecting ARPE-19 cells from NaIO_3_-induced cell death (Figure 2(f)). Moreover, pretreatment with Zileuton also mitigated ferroptosis induced by conventional ferroptosis inducers including RSL3 (1 *μ*M), Erastin (10 *μ*M), and combined treatment with FeSO_4_ (100 *μ*M) and tBHP (250 *μ*M). The role of 5-LOX in ferroptosis was verified by the observed resistance to Erastin-induced cell death in *ALOX5*-KD ARPE-19 cells (Figure 3). These findings suggest the significance of 5-LOX in ferroptosis. Inhibition of 5-LOX could be a beneficial approach to protect RPE from oxidative stress-induced cell death.

**Figure 4:**
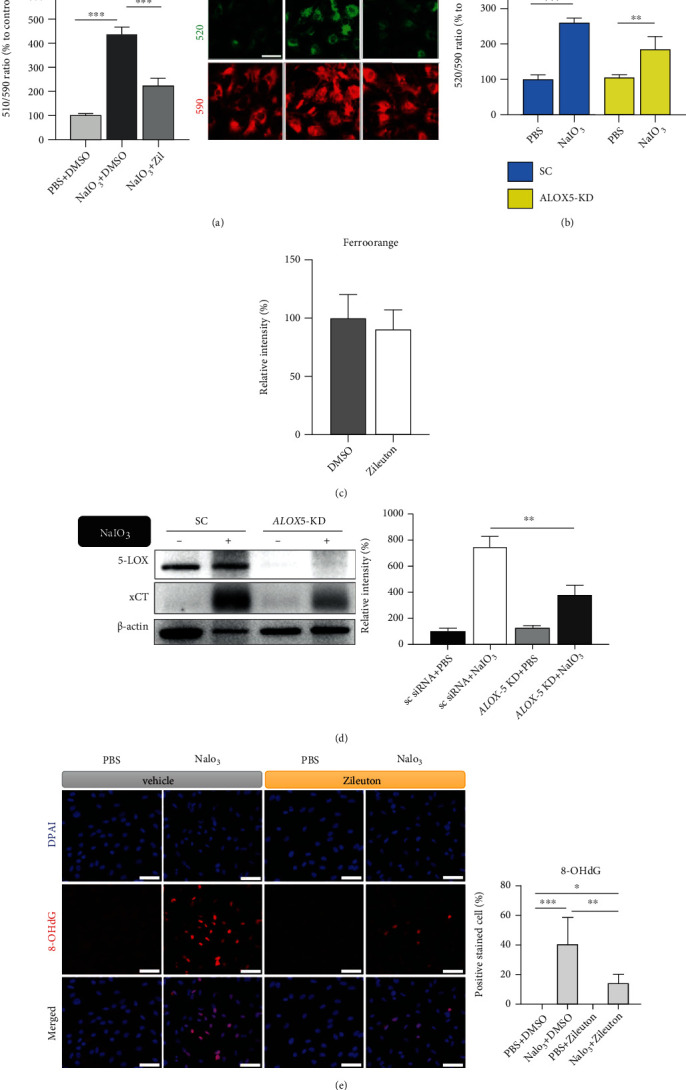
Inhibition of lipoxygenase-5 (5-LOX) reduced NaIO_3_-induced oxidative stress in ARPE-19 cells. (a, b) BODIPY C11 staining lipid peroxidation (*n* = 6, 35 mM NaIO_3_,15 h, Zileuton 75 *μ*M). (c) FerroOrange staining for intracellular ferrous ions (75 *μ*M Zileuton, *n* = 12). (d) Ferroptosis-associated protein xCT detected with western blot analysis (18 mM NaIO_3_ for 15 h, *n* = 3). (e) Oxidative stress-induced DNA damage demonstrated with 8-OHdG staining (*n* = 4, 35 mM NaIO_3_, 75 *μ*M Zileuton, 15 h). Scale bar = 50 *μ*m. Abbreviations: xCT: cystine-glutamate antiporter; 8-OHdG: 8-hydroxy-2′-deoxyguanosine; Zil: Zileuton.

Lipid peroxidation is one of the key players in ferroptosis. Pretreatment with Zileuton and knockdown of *ALOX5* both reduced NaIO_3_-induced green fluorescence emission (520 nm) of BODIPY C11, an indicator of lipid peroxidation, in live ARPE-19 cells (Figures 4(a) and 4(b)). Intracellular iron level may affect the susceptibility of cells to ferroptosis. However, FerroOrange staining showed that treatment with Zileuton (75 *μ*M) for 24 h did not affect the level of intracellular ferrous iron (Figure 4(c)). NaIO_3_ induced the upregulation of ferroptosis-associated antioxidant protein cystine-glutamate antiporter (xCT or SLC7A11) that is involved in the intracellular transport of cysteine and glutathione production. Furthermore, the upregulation of xCT was alleviated in *ALOX5*-KD ARPE-19 cells, which suggests lower levels of ROS (Figure 4(d)). Zileuton pretreatment mitigated the percentage of 8-OHdG-positive nuclei, an indicator of oxidative stress-induced DNA damage, in ARPE-19 cells (Figure 4(e)). These results suggest that inhibition of 5-LOX suppresses NaIO_3_-induced ROS, which may be correlated with cell death in RPE.

### 3.3. NaIO_3_ Induces Mitochondrial Damage in ARPE-19 Cells

**Figure 5:**
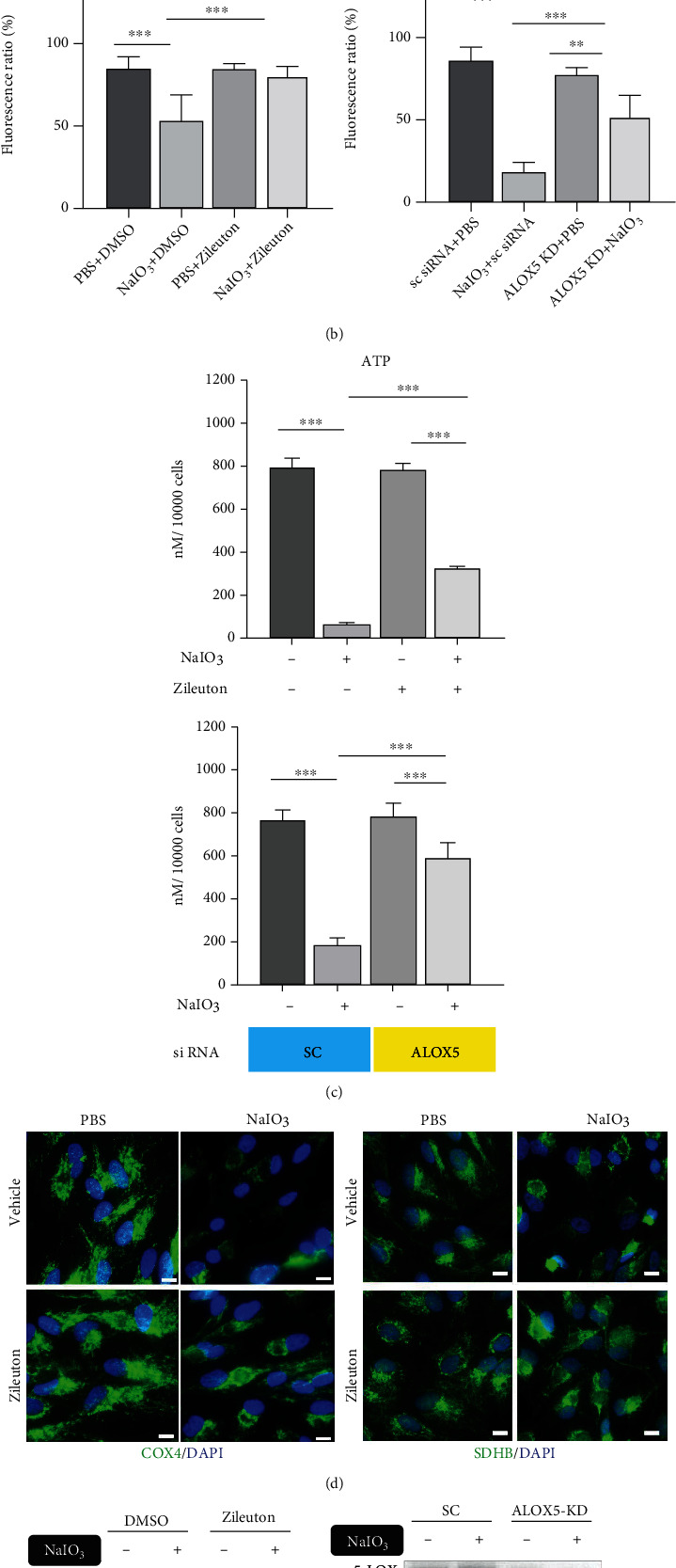
NaIO_3_ induced mitochondrial damage, which was mitigated with LOX-5 inhibition in ARPE-19 cells. (a) Mitochondrial ROS staining with MitoSOX (NaIO_3_ 35 mM,15 h, *n* = 12). (b) Mitochondrial transmembrane potential detected with JC-1 staining (NaIO_3_ 35 mM, 15 h, *n* = 6). (c) ATP production (NaIO_3_ 35 mM, 15 h, *n* = 6). (d) Representative immunofluorescence staining for mitochondrial protein COX-4 and SDHB (NaIO_3_ 35 mM, 15 h, *n* = 6). Scale bar = 10 *μ*m. (e) The loss of mitochondrial mass was evaluated with western blot analysis of SDHA. Abbreviations: COX-4: cytochrome c oxidase subunit 4; SDHA: succinate dehydrogenase complex, subunit A, flavoprotein variant; SDHB: succinate dehydrogenase [ubiquinone] iron-sulfur subunit, mitochondrial.

Mitochondria contain membranous structures and iron and therefore may be a target of oxidative damage in ferroptosis [20, 30, 31]. NaIO_3_ treatment caused an increase in mitochondrial ROS, detected with MitoSOX staining (Figure 5(a)). Additionally, a decrease in mitochondrial membrane potential (*ΔΨ*m) was detected with JC-1 staining (Figure 5(b)), along with a decrease in ATP production (Figure 5(c)). Pretreatment with Zileuton (75 *μ*M) alleviated NaIO_3_-induced mitochondrial ROS, decreased *ΔΨ*m and ATP production, and decreased expression of mitochondrial proteins including COX-4, SDHB, and SDHA, demonstrated by immunofluorescence imaging or western blot analysis (Figures 5(d) and 5(e)). Using *ALOX5*-KD ARPE-19 cells, the role of 5-LOX was further verified by rescue of impaired *ΔΨ*m, ATP production, and downregulated mitochondrial proteins (Figures 5(b), 5(c), and 5(e)).

### 3.4. NaIO_3_ Caused RPE Loss, Inflammatory Responses, and Damage of Mouse Retina

**Figure 6:**
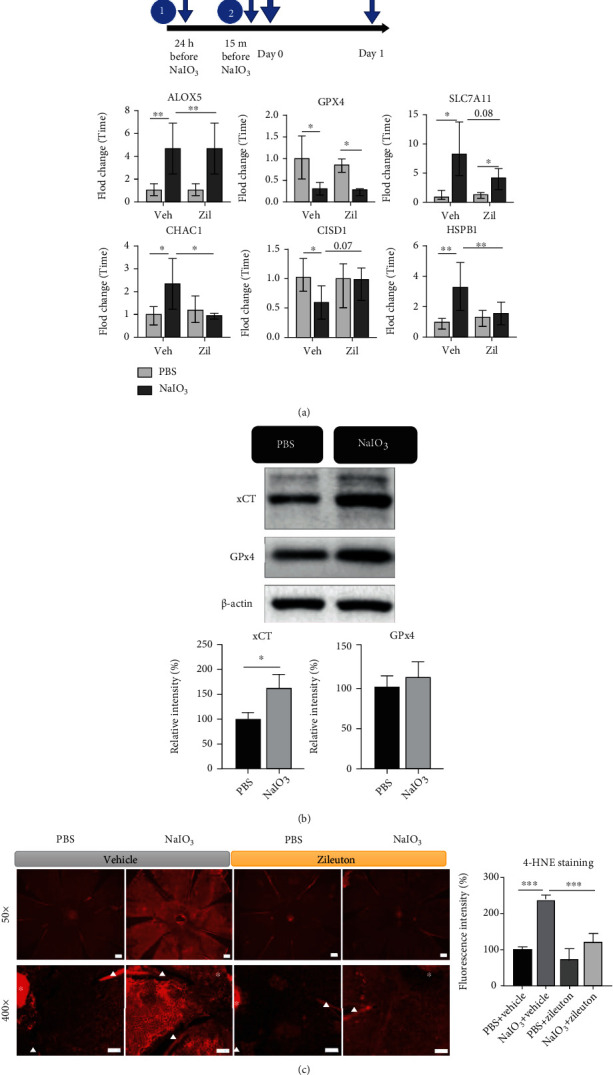
NaIO_3_ (40 mg/kg) induced lipid peroxidation in the RPE monolayer and cell death of photoreceptors after 24 h. (a) mRNA expression of ferroptosis-associated genes in RPE/choroid assessed by real-time PCR (*n* = 6 per treatment condition). (b) xCT and GPx4 protein expression in RPE/choroid assessed by western blot analysis (*n* = 3 per treatment condition). (c) Immunofluorescence staining of 4-hydroxynonenal (4-HNE) in RPE monolayer (*n* = 4 per treatment condition). Triangle: radial cut of flat mount; asterisks: optic nerve. Scale bar = 200 *μ*m in the upper row and 100 *μ*m in the lower row. Abbreviations: veh: vehicle; Zil: Zileuton.

**Figure 7:**
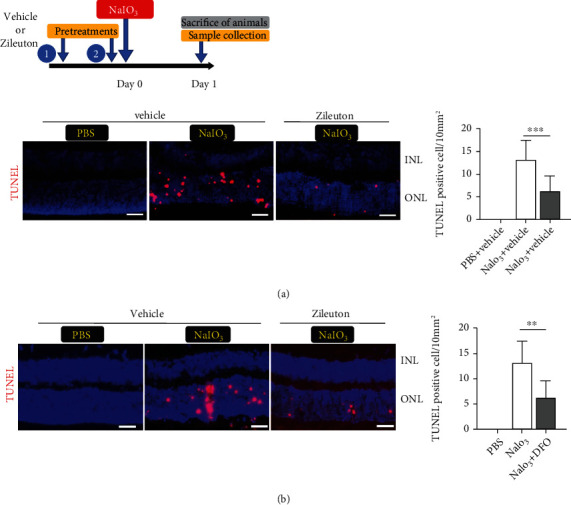
NaIO_3_ (40 mg/kg) induced photoreceptor cell death after 24 h. The effect of (a) Zileuton and (b) DFO on cell death was assessed by TUNEL staining (red, *n* = 8) in the outer retinal layer (ONL). Scale bar = 50 *μ*m. Abbreviations: DFO: deferoxamine; INL: inner nuclear layer; ONL: outer nuclear layer of the retina.

To verify the effects of NaIO_3_ and the role of 5-LOX in RPE in vivo, we used a murine model of NaIO_3_ (intraperitoneal injection 40 mg/kg body weight)-induced acute retinal degeneration using 6-week-old male wild-type C57BL/6JNarl mice. To detect ferroptosis after NaIO_3_ treatment in vivo, we examined genes associated with ferroptosis using real-time PCR. Changes in the mRNA expression of ferroptosis-associated genes *ALOX5*, *GPX4*, *SLC7A11*, *CHAC1*, *CISD1*, and *HSPB1* were observed in RPE/choroid samples collected 1 day after NaIO_3_ treatment (Figure 6(a)). Unexpectedly, we observed a decrease in mRNA expressions of *GPX4* and *CIS1D*, previously reported to protect mitochondria from lipid peroxidation [32], following the NaIO_3_ treatment. NaIO_3_-induced change in the protein level of xCT (SLC7A11) was consistent with change in mRNA in RPE/choroid (Figure 6(b)). Pretreatment with Zileuton (20 mg/kg intraperitoneal, 24 h and  15min before NaIO_3_ injection) modulated NaIO_3_-induced changes in mRNA expression including *ALOX5*. These results suggest the potential participation of 5-LOX in cell death such as ferroptosis induced by NaIO_3_ in vivo. NaIO_3_ induced lipid peroxidation as evidenced by positive staining for 4-HNE, particularly in the membrane of cells in the RPE monolayer (Figure 6(c)). Meanwhile, cell death, demonstrated by positive TUNEL staining, was observed in the retinal photoreceptor layer 24 h after NaIO_3_ treatment (Figure 7(a)). Pretreatment with Zileuton effectively reduced NaIO_3_-induced cell death in the retina and 4-HNE staining of RPE (Figures 7(a) and 6(c)). Induction of ferroptosis was supported by the protective effect of DFO, which reduced NaIO_3_-induced cell death in mouse retina (Figure 7(b)).

**Figure 8:**
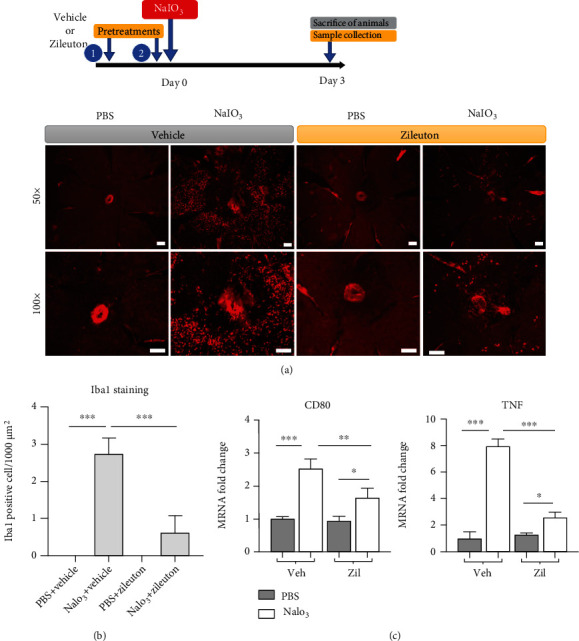
NaIO_3_ (40 mg/kg) induced inflammatory response in RPE monolayer after 3 days. (a) Representative immunofluorescence images of Iba1 staining in RPE monolayer of mice (50x upper and 100x lower rows, centered at the optic nerve). Scale bar = 200 *μ*m. (b) Quantification of Iba1-positive cells (*n* = 4 per treatment condition). (c) mRNA expression of inflammatory genes *CD80* and *TNF* in cells derived from the eyecup including the RPE monolayer assessed by real-time PCR (*n* = 6 per treatment condition). Abbreviations: Iba1: ionized calcium-binding adapter molecule 1; Zil: Zileuton.

**Figure 9:**
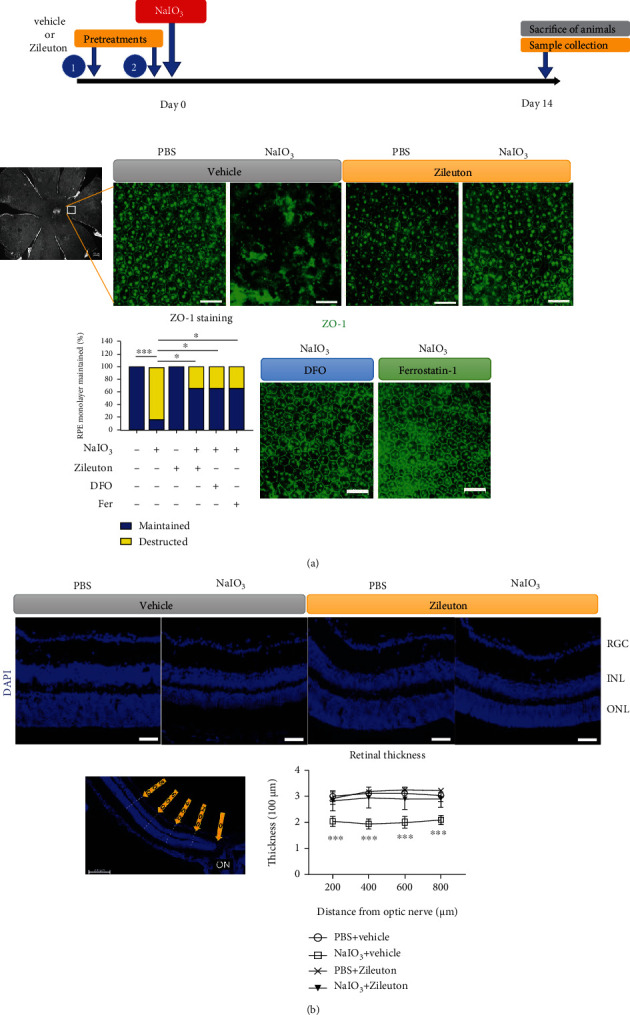
NaIO_3_ (40 mg/kg) induced destruction of RPE monolayer and thinning of the retina after 2 weeks. (a) Immunofluorescence staining of ZO-1 in the RPE monolayer of mice (*n* = 12 per treatment condition). (b) Retinal thickness was measured at 200, 400, 600, and 800 *μ*m from the optic disc in DAPI-stained retinal cross sections (*n* = 6). Scale bar = 50 *μ*m. Abbreviations: DFO: deferoxamine; Fer: ferrostatin-1; ON: optic nerve; ZO-1: zonula occludens-1; DAPI, 4′,6-diamidino-2-phenylindole.

Infiltration of inflammatory cells in the retina and/or RPE layers can be related to cell death. An increasing number of active microglia or monocytic cells with amoebic, round, or oval-shaped and positive Iba1-staining were observed in the RPE monolayers 3 days following NaIO_3_ treatment (Figures 8(a) and 8(b)). Pretreatment of mice with Zileuton reduced the number of Iba1-positive cells on the RPE monolayer surface and downregulated the expression of proinflammatory genes, including *CD80* and *TNF* in the cells derived from the RPE and eyecup (Figure 8(c)). Destruction of RPE monolayer integrity was observed 14 days following NaIO_3_ treatment in mice. Pretreatment with Zileuton protected the RPE monolayer from NaIO_3_-induced destruction, which was demonstrated with ZO-1 staining. Pretreatment with ferroptosis inhibitors, such as DFO and ferrostatin-1, showed similar protective effects in the RPE monolayer (Figure 9(a)). This finding also supported the involvement of cell death mechanisms related to ferroptosis in NaIO_3_-induced destruction of RPE *in vivo*. Additionally, NaIO_3_ induced reduction in the thickness of neuroretina, which was also mitigated with Zileuton (Figure 9(b)).

## 4. Discussion

**Figure 10:**
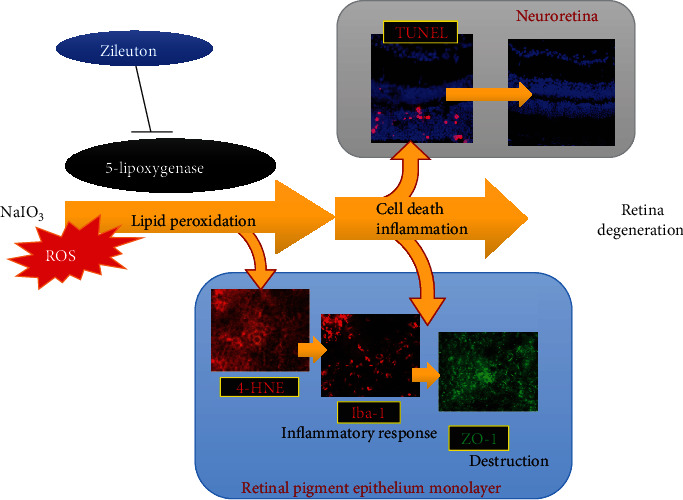
Schematic summary of the role of 5-lipoxygenase in ROS-induced retinal degeneration.

In this study, NaIO_3_-induced lipid peroxidation and cell death were mitigated with the 5-LOX inhibitor Zileuton in ARPE-19 cells (Figures 2 and 4). Furthermore, the potential benefit of 5-LOX inhibition was validated for the first time in an *in vivo* model of NaIO_3_-induced acute retinal degeneration. Inhibition of 5-LOX decreased lipid peroxidation of RPE cells (Figure 6), cell death in the photoreceptor layer of mouse retina (Figure 7), activation of inflammatory cells (Figure 8), and degeneration of the neuroretina and RPE monolayer following NaIO_3_ treatment (Figure 9). Pretreatment with ferroptosis inhibitors including DFO and ferrostatin-1 exerted similar effects in protecting RPE cells from NaIO_3_-induced damage both in *in vitro* and *in vivo* models (Figures 1, 2, and 9). Additionally, the role of 5-LOX in cell death caused by conventional ferroptosis inducers, such as Erastin was demonstrated *in vitro* (Figure 3). Our results suggest that ferroptosis was one of the major mechanisms involved in oxidative stress-induced cell death and that 5-LOX was a crucial enzyme that participated in the ROS-induced response and induction of ferroptosis in RPE cells. Therefore, modulating 5-LOX activity could be a promising strategy to alleviate damages induced by accumulation of ROS and disturbance of iron homeostasis in retinal diseases, such as AMD. The graphic summary of the proposed mechanisms is presented in Figure 10.

Research on ferroptosis in the retina is still in its early stages. Ferroptosis has been implicated to be one of the mechanisms associated with stress-induced cell death and senescence in RPE [10–12, 33, 34]. The importance of antioxidative enzyme systems in the protection of retina degeneration has been emphasized previously. Increased expression of GPx4 provided strong functional and structural protection from oxidative damage in a transgenic mouse model [35]. Iron is essential for energy generation and metabolism in most tissues involved in energy homeostasis [36]. However, it has been reported that genetic iron-overload in diabetic Hfe-knockout mice escalated neuronal cell death, vascular damages, and defects in retinal barrier integrity [37]. Apart from DR, iron may also be involved in a broad range of retinal disorders. It has been reported that the iron levels in the human macula increase with age. Postmortem retinal tissues derived from patients with AMD had higher iron and iron-carrying transferrin protein (TF) than age-matched controls [15]. The increase in both iron and TF has been shown to be correlated with poor visual acuity in murine models of retinal detachment. Exogenous transferrin can thus help in iron clearance to decrease cell death induced by retinal detachment [38]. Ferrous ions have also been found to interact with bis-retinoids in the RPE and promote cell damage [39]. In another study, iron-chelating reagent metallocomplex zinc-desferrioxamine lowered lipid peroxidation and oxidative DNA damage in a murine model of retinitis pigmentosa [40]. These findings suggest that iron metabolism potentially participates in cell death observed in retinal diseases.

In addition to abnormal iron metabolism, research on ferroptosis-associated molecules and signaling pathways has also increased. Metabolism of cysteine, iron and lipid, mevalonate pathway, GPx4 activity, and autophagy have been reported to participate in the regulation of ferroptosis [20, 31, 41]. However, simple blockage of these metabolic and signaling pathways can cause disturbance and unexpected impairment in retinal functions; therefore, identification of a feasible treatment to control ferroptosis is challenging. In this study, NaIO_3_ induced changes in the mRNA expression of ferroptosis-associated genes including *ALXO5*, *GPX4*, *SLC7A11*, *CHAC1*, and *HSPB1* in the mouse RPE/eyecup tissue (Figure 6) [32, 42, 43]. The understanding of 5-LOX activity in the RPE or retina is limited. We found that the protective effect of 5-LOX inhibition was mostly associated with NaIO_3_-induced oxidative stress, especially lipid peroxidation, and less likely associated with modulation of intracellular iron levels in ARPE-19 cells (Figure 4). It has previously been reported that DHA suppressed hydrogen peroxide-induced transcriptional upregulation of 5-LOX and associated proinflammatory HETEs, following omega-6 PUFA oxidation observed in ARPE-19 cells [25]. Some recombinant PEDF-R proteins, which can inhibit 5-LOX activity, have been shown to protect RPE cells from ROS-induced cell death *in vitro* via the inhibition of leukotriene production [24]. LOXs including 5-12- and 15-LOX can metabolize arachidonic acid and PUFA into inflammatory chemokines including leukotrienes and prostaglandins to counteract with the damages caused in diseases [44, 45]. Nevertheless, it has been reported that downstream metabolites of LOXs, such as HPETEs, can react with ROS and contribute to the lipid peroxidation, which induces ferroptosis. Mobilization of arachidonic acid under oxidative stress has also been reported [28]. In this study, we found that NaIO_3_ upregulated both arachidonic acid and 5-HETE in ARPE-19 cells (Figure 2). This could indicate the possibility of transient increase of unstable hydroperoxide derivatives, such as 5-HPETE, which may react with ROS to cause lipid peroxidation of cellular membrane structures through Fenton reaction inducing ferroptosis [21, 22]. In addition to cell death, NaIO_3_ caused a significant increase in the density of inflammatory cells in the RPE monolayer of the eyecup (Figure 8). ROS-damaged RPE cells potentially release proinflammatory signals, such as leukotriene B4, which can lead to infiltration of inflammatory cells in the retina [24]. However, the exact role of 5-LOX inhibition in the differentiation of inflammatory cells was not explored in this study and requires further investigation.

Upregulation of antioxidant proteins including xCT (SLC7A11) *in vitro* (Figure 4) and *SCL7A11* and *CHAC1* mRNA *in vivo* (Figure 6) suggested the activation of the intracellular antioxidant system, which utilizes glutathione to counteract NaIO_3_-induced increase in ROS. Upregulation of xCT was observed in both i*n vitro* and *in vivo* experiments, suggesting that xCT may be a sensitive marker of ROS in RPE cells. This can also indicate shortage of cysteine following oxidative stress, and induction of ferroptosis in cysteine deprivation condition [41]. In addition, we found that NaIO_3_ induced significant loss of ATP production, mitochondrial transmembrane potential, and expression of mitochondrial proteins essential for citric acid cycle and the electron transport chain (Figure 5). Mitochondria are iron-rich organelles, in which iron is incorporated to form iron–sulfur clusters in the electron transport chain. However, abnormality in iron metabolism, particularly ferrous ions or excessive ROS, can induce lipid peroxidation through Fenton reaction, which may compromise mitochondrial membrane integrity and subsequently induce bioenergetic crisis and cell death [20]. This is of particular significance in the retina, which is a tissue with high metabolic demand and rich in mitochondria. In this study, Zileuton prevented an increase in ROS and mitigated the loss of mitochondrial proteins including SDHA, SDHB, and COX4, thereby preserving ATP production in ARPE-19 cells (Figure 5). Consistent with our findings, 5-LOX was previously shown to mediate mitochondrial damage and cell death in mononuclear cells in end-stage retinal disease patients and PC-12 neuronal cells [46]. NaIO_3_-induced drop in *CIS1D* mRNA, which regulates iron homeostasis in mitochondria, may indicate the involvement of mitochondria in ferroptosis in RPE cells *in vivo*. Overall, our results support the hypothesis that mitochondria are implicated in ferroptosis, and inhibition of 5-LOX can partially preserve mitochondrial integrity and function in RPE cells.

Our results suggested that NaIO_3_-induced oxidative damage, although a fast model to investigate the effects of ROS in the retina and RPE, should be interpreted cautiously. NaIO_3_ may induce cell death through multiple mechanisms besides ferroptosis in the RPE *in vivo*, and it is not easy to exclude the possibility that 5-LOX is activated by mechanisms other than NaIO_3_-induced ferroptosis in the retina. Nevertheless, NaIO_3_-induced ferroptosis was confirmed through the protective effects of the iron chelator, DFO, which inhibited cell death in the outer retinal layer and destruction of the RPE monolayer. Additionally, cell death induced by conventional ferroptosis inducers Erastin and RSL3 was successfully blocked via 5-LOX inhibition with Zileuton in ARPE-19 cells, providing evidence for the role of 5-LOX in ferroptosis in RPE cells *in vitro*. Moreover, combination of Zileuton with DFO did not exert additive effects when compared with Zileuton alone. Taken together, these results suggest that 5-LOX inhibition can effectively block ferroptosis in NaIO_3_-injured RPE.

## 5. Conclusions

In summary, we demonstrated the involvement of 5-LOX in oxidative stress in RPE cells, and that 5-LOX inhibition protected RPE cells from NaIO_3_-induced lipid peroxidation, mitochondrial damage, DNA damage, and cell death. The observed increase in 5-HETE provided evidence for the activation of the 5-LOX pathway and increase in the upstream unstable 5-HPETE. These eicosanoids can react with ROS to cause lipid peroxidation of cellular membrane structures ultimately leading to cell death through mechanisms including ferroptosis. A novel finding of our study was the protective effect of 5-LOX inhibition on RPE and retina from ROS-induced inflammation and cell death *in vivo*. Our results highlight the potential of 5-LOX inhibition as a feasible strategy for control of ROS damage in RPE and retina in retinal diseases, such as AMD. Future studies with genetic and pharmaceutical approaches are required to confirm the role of 5-LOX activity in managing retinal diseases associated with abnormal iron metabolism and oxidative stress.

## Figures and Tables

**Table 1:** Primers of mouse genes for real-time PCR.

Gene name	Accession number	Forward primer	Revere primer
ALOX5	NM_009662	TCTTCCTGGCACGACTTTGCTG	GCAGCCATTCAGGAACTGGTAG
GPX4	NM_001037741	CCTCTGCTGCAAGAGCCTCCC	CTTATCCAGGCAGACCATGTGC
SLC7A11	NM_011990	CTTTGTTGCCCTCTCCTGCTTC	CAGAGGAGTGTGCTTGTGGACA
CHAC1	NM_026929	TGACCCTCCTTGAAGACCGTGA	AGTGTCATAGCCACCAAGCACG
CISD1	NM_134007	AAGACAACCCGAAGGTGGTGCA	CTTCGTTGTGCTTTATGTGAGCC
HSPB1	NM_013560	GCTCACAGTGAAGACCAAGGAAG	TGAAGCACCGAGAGATGTAGCC
CD80	NM_009855	CCTCAAGTTTCCATGTCCAAGGC	GAGGAGAGTTGTAACGGCAAGG
TNF	NM_013693	GGTGCCTATGTCTCAGCCTCTT	GCCATAGAACTGATGAGAGGGAG
ACTB	NM_007393	CGGTTCCGATGCCCTGAGGCTCTT	CGTCACACTTCATGATGGAATTGA

The primers for *ALOX5*, *GPX4*, *SCL7A11*, *CHACI*, *CISDI*, *HSPB1*, *CD80*, and *TNF* were from OriGene (Rockville, MD, USA).

## Data Availability

Data is contained within the article or the supplemental material.

## Supplementary Materials

Supplementary Materials Method S1: detection of intracellular arachidonic acid and its metabolites.
