# Supplementary material for: 5-Lipoxygenase Inhibition Protects Retinal Pigment Epithelium from Sodium Iodate-Induced Ferroptosis and Prevents Retinal Degeneration
Source: Oxid Med Cell Longev. 2022 Feb 23;2022:1792894. doi: 10.1155/2022/1792894 (PMC8890867; doi:10.1155/2022/1792894)
Supplement: Supplementary Materials — Method S1: detection of intracellular arachidonic acid and its metabolites. [file 1792894.f1.docx]

Detection of intracellular arachidonic acid and its metabolites

1. **Experimental**

**1.1. Materials**

# 5-Hydroxyeicosatetraenoic acid(5-HETE), 8-iso Prostaglandin F2α (8-iso PGF2α), Prostaglandin F2α (PGF2α), Arachidonic acid (ARA), 5-Hydroxyeicosatetraenoic acid-D_8_(5-HETE-D_8_), 8-iso Prostaglandin F2α-D_4_(8-iso PGF2α-D_4_), Prostaglandin F2α-D_4_(PGF2α-D_4_), Arachidonic acid-D_8_(AA-D_8_) were purchased from Cayman Chemicals. Deuterated compounds (5-HETE-D_8_, 8-iso PGF2α-D_4_, PGF2α-D_4_ and AA-D_8_) were used as internal standards for quantification. Acetonitrile (HPLC-grade) and Methanol (HPLC-grade) were purchased from Sigma-Aldrich (St. Louis, MO, USA), Ethyl Acetate (HPLC-grade) were purchased from Duksan Techopia Co., Ltd (Cheonan, Korea). Formic acid (ACS grade) was purchased from Honeywell Company (Charlotte, NC, USA). Ultrapure water was from Milli-Q Advantage A10 system (Merck Millipore, Burlington, MA, USA).

**1.2. Preparation of standards and quality control (QC) solution**

Stock solutions of all analytes were prepared in methanol at a concentration of 2 μg/ml for 5-HETE and PGF2α-D_4_ , 5 μg/ml for 5-HETE-D_8_ and 8-iso PGF2α-D_4_, and 100 μg/ml for all other analytes. Mix stocks of analytes and internal standards were prepared in Acetonitrile and 0.1% formic acid aqueous solution (1:1, v/v) at a concentration of 1 μg/ml.

Standard solutions were prepared in Acetonitrile and 0.1% formic acid aqueous solution (1:1, v/v) by spiking with an appropriate volume of the serially diluted mix stock solutions, resulting in different concentrations required for the calibration curve (0.5~500 ng/ml). Quality control (QC) samples were prepared daily at two different concentration levels including low and high concentrations (1.0 and 5.0 ng/ml).

**1.3. Standards, Quality control and Sample preparation**

For preparation, 20 μl of internal standards mix stocks**,** 0.5 ml of 1% formic acid aqueous solution

were added to 100 μl of sample, then extract with 3 ml of ethyl acetate and centrifuge at 4000 rpm for 5 minutes. the supernatant was dried under nitrogen stream and reconstitute with 50 μl acetonitrile and 50 μl of 0.1% formic acid aqueous solution. Finally, aliquot of 15 μl of the residues was injected into the LC–MS/MS system for analysis. The preparation of Standards and Quality control solution were the same as the sample.

- 1. **LC-MS/MS analysis**

Chromatography was performed using an Agilent 1200 Infinity II HPLC system equipped with a Agilent Poroshell EC-18 column (150 mm × 3.0 mm, 2.7 μm). The components were eluted by a gradient of (A) Ultrapure water and (B) Acetonitrile. The gradient conditions of the mobile phase over 23 min were as follows: 0–4 min, 40-45% B; 4–5 min, 45–90% B; 5–7 min, 90-95% B; 7–16 min, 95% B; 16–16.1 min, 95-40% B and 16.1–23 min, 40% B. The flow rate was 0.3 mL/min. Column oven was 40℃. The autosampler was maintained at 4 °C throughout the analyses. Agilent 1200 Infinity II HPLC system was coupled with an Agilent 6470B Triple Quadrupole LC/MS^2^. Negative ionization mode for electrospray ionization source were used for detection. Data were collected in multiple reaction monitoring (MRM) Mode. The mass transitions, Fragmentor (FP) and collision energy (CE) for the analytes are described in Table S1. The data were acquired and processed using Agilent MassHunter Workstation Version 10.1 software.

Table S1: Multiple reaction monitoring (MRM) parameters for ARA and its metabolites.

| Compound name | Precursor ion  (m/z) | Product ion  (m/z) | Fragmentor  (V) | Collision energy(eV) | [Retention Time](https://terms.naer.edu.tw/detail/1318638/)  (min) | Internal standard |
| --- | --- | --- | --- | --- | --- | --- |
| 5-Hydroxyeicosatetraenoic acid (5-HETE) | **319**  319 | **301**  257 | 90  90 | 8  8 | 11.0 | 5-HETE-D_8_ |
|  |  |  |  |  |  |  |
| 8-iso Prostaglandin F2 alpha  (8-iso PGF2α ) | **353**  353 | **309**  291 | 145  145 | 20  20 | 4.4 | 8-iso PGF2α-D_4_ |
|  |  |  |  |  |  |  |
| Prostaglandin F2 alpha  (PGF2 α) | **353**  353 | **309**  291 | 130  130 | 16  20 | 5.2 | PGF2α-D_4_ |
|  |  |  |  |  |  |  |
| Arachidonic acid  (ARA) | **303**  303 | **259**  205 | 130  130 | 8  12 | 14.2 | AA-D_8_ |
|  |  |  |  |  |  |  |
| 5-HETE-D_8_ | **327** | **309** | 105 | 8 | 11.0 |  |
|  | 327 | 265 | 105 | 12 |  |  |
| 8-iso PGF2α-D_4_ | **357** | **313** | 150 | 20 | 4.4 |  |
|  | 357 | 197 | 150 | 28 |  |  |
| PGF2α-D_4_ | **357** | **313** | 130 | 20 | 5.2 |  |
|  | 357 | 197 | 130 | 24 |  |  |
| ARA-D_8_ | **311** | **267** | 110 | 12 | 14.2 |  |
|  | 311 | 212 | 110 | 12 |  |  |

ARA

8-iso PGF2α

5-HETE

PGF2α

Fig. 1. Total ion chromatogram for ARA and its metabolites


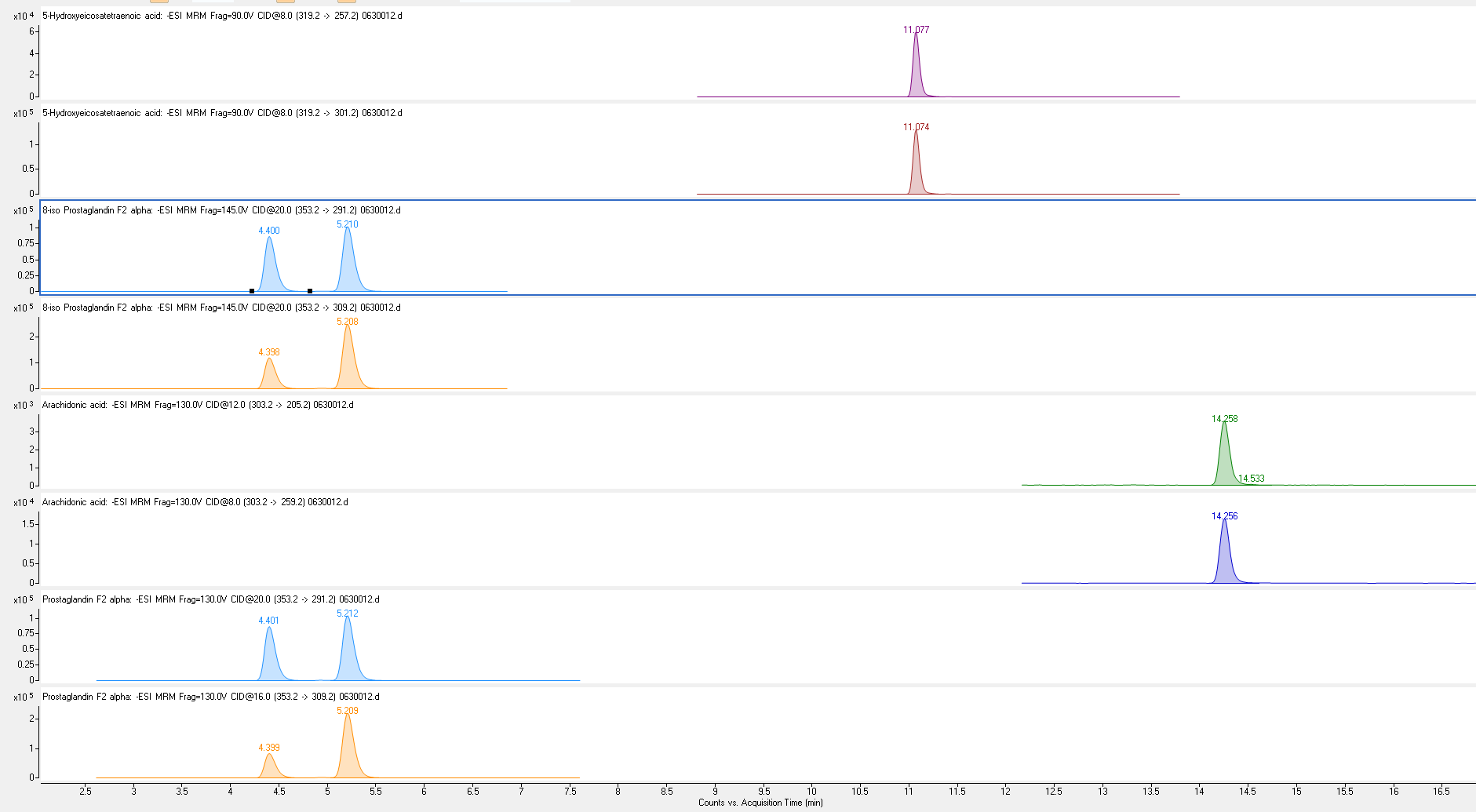


ARA

ARA

353＞309 m/z

353＞291 m/z

303＞259 m/z

303＞205 m/z

353＞291 m/z

353＞309 m/z

319＞301 m/z

319＞257 m/z

5-HETE

5-HETE

PGF2α

PGF2α

8-iso PGF2α

8-iso PGF2α

Fig. 2. Multiple reaction monitoring (MRM) chromatograms for ARA and its metabolites.
